# NRF-1 transcription factor regulates expression of an innate immunity checkpoint, CD47, during melanomagenesis

**DOI:** 10.3389/fimmu.2024.1495032

**Published:** 2024-12-17

**Authors:** Kuldeep Makwana, Edwin J. Velazquez, Diego M. Marzese, Bethany Smith, Neil A. Bhowmick, Mark B. Faries, Omid Hamid, Alexander D. Boiko

**Affiliations:** ^1^ Department of Medicine, Comprehensive Cancer Institute, Cedars-Sinai Medical Center, Los Angeles, CA, United States; ^2^ Department of Surgery, Duke University School of Medicine, Durham, NC, United States; ^3^ Cancer Epigenetics Laboratory, Health Research Institute of the Balearic Islands (IdISBa), Palma, Spain; ^4^ The Angeles Clinical and Research Institute, a Cedars-Sinai Affiliate, Los Angeles, CA, United States; ^5^ Department of Biomedical Sciences, Comprehensive Cancer Institute, Cedars-Sinai Medical Center, Los Angeles, CA, United States

**Keywords:** cd47, NRF1, melanoma, promoter regulation, immune checkpoint

## Abstract

Transmembrane integrin-associated protein *CD47* functions as a potent innate immunity checkpoint and is upregulated by many types of malignant cells, including melanoma during tumor progression. Binding of *CD47* to its target receptor, SIRPα, on myeloid cell lineages leads to the initiation of the downstream signaling cascades that inhibit innate immunity anti-tumor responses. Molecular mechanisms underlying upregulation of *CD47* during melanoma progression remain largely unknown. In this report, we performed ATAC-Sequencing on patient-derived melanoma cells, as well as, the analysis of ATAC-Seq datasets covering clinical melanoma samples to demonstrate a significant increase in chromatin accessibility for the *CD47* promoter region in comparison to normal cells and tissues. Additionally, profiling of multiple *CD47* transcript isoforms established that upregulation of *CD47* in malignant cells occurs at the mRNA level. Using chromatin immunoprecipitation (ChIP) approaches along with the analysis of ChIP-Seq cancer datasets, we identified the transcription factor NRF-1 which binds at multiple sites within the proximal *CD47* promoter region. In combination with serial deletions of *CD47* promoter, we defined the minimal DNA region required for its activation, as well as, specific DNA locations within that region, which are preferentially occupied by NRF-1 in tumor cells.

## Introduction

Understanding mechanisms leading to the acquisition of immune-evasive properties by malignant cells represents a critical step in the design of effective anti-cancer regimens. Previous studies by our group and others identified integrin-associated protein *CD47* as the critical myeloid lineage checkpoint that is overexpressed by various types of tumor cells, including melanoma, and inhibits innate immunity-mediated anti-tumor responses (1–4). First discovered on the cell surface of circulating red blood cells (RBCs), *CD47* is able to interact with the SIRPα receptor present on the macrophages to inhibit their phagocytic function, a phenomenon that later became known as the “don’t eat me” signal (5). Subsequent research on hematopoietic malignancies including acute myeloid leukemia (AML) and non-Hodgkin lymphoma (NHL) revealed that malignant cells hijack this mechanism to evade surveillance by an innate immunity during cancer progression (4, 6, 7). These results were later extended into the studies of the solid tumors, where it was shown that high levels of *CD47* are associated with disease progression and/or poor patient prognosis, including epithelial (breast, ovarian, colon, and others) (8–10), mesenchymal (sarcomas) (11, 12), and neuronal/neural crest (GBM, Neuroblastoma, Melanoma) derived malignancies (2, 13). Importantly, past research by our group demonstrated that freshly resected metastatic human melanoma tumors are highly positive for *CD47*, while its blockade (by administering inhibitory antibodies) led to the significant suppression of melanoma metastases *in-vivo* using several independently patient-derived xenografts (PDXs) (2). Clinical significance of these findings has been translated into the development of therapeutic compounds blocking *CD47* interaction with SIRPα in order to reinvigorate anti-tumor immune responses, with some of them reaching Phase I and II human trials (14, 15).

While the immune-suppressive role of *CD47* on the cell surface of tumor cells has been characterized to a great extent, mechanisms underlying its regulation during human cancer progression are only beginning to emerge. These studies reveal substantial variability in the transcription factor machinery involved in the regulation of the *CD47* promoter depending on the tumor type. Thus, it has been shown that in leukemias (both human and mouse) MYC oncogene binds to the distal enhancer regions of the *CD47* promoter to regulate its activity (16), while HIF-1α and NFκB transcription factors have been implicated in the regulation of *CD47* promoter in breast, cervical, and non-small lung carcinoma cells (17–19).

In this report, we find that *CD47* upregulation in melanoma occurs at the mRNA level and is associated with changes in chromatin accessibility at the *CD47* promoter region using the assay for transposase-accessible chromatin with sequencing (ATAC-seq). This leads to an increased *CD47* mRNA production, as determined by real-time PCR covering multiple transcript variants of this gene. To reveal molecular factors regulating *CD47* mRNA expression we performed an extensive analysis of the *CD47* promoter region using the MotifMap algorithm. As a result, we identified multiple locations containing the binding consensus sequence for the Nuclear Respiratory Factor-1 (NRF-1) within the proximal promoter region of the *CD47* gene. Furthermore, Chromatin Immunoprecipitation (ChIP) assays revealed NRF-1 to be bound at multiple proximal *CD47* promoter sites with differential affinity among malignant and normal cell types leading to increased *CD47* mRNA and protein levels. Lastly, using bioluminescence reporter systems, we were able to define the minimum *CD47* promoter region and the number of NRF-1 binding sites required for its efficient activation.

## Materials and methods

### Cell culture

Melanoma cells M1626 and M727 were maintained in RPMI with 10% FBS, M525 cells were maintained in RPMI with 5% FBS, HepG2 cells were maintained in DMEM with 10% FBS, and M354 cells were maintained in 1:1 of RPMI with 10% FBS and dermal cell basal media from ATCC (PCS-200-030). Normal melanocytes were maintained in dermal cell basal media from ATCC (PCS-200-030). All cells were grown in Tissue Culture incubators set at 5% CO2 and 37°C degrees. For NRF1 siRNA mediated knockdown, a total of 1 × 10^5^ M727 cells were seeded in 6 well plates and incubated overnight at 37 ° C and 5% CO_2_. Cells were then transfected using 8 μl of siRNA transfection reagent (Santa Cruz, Cat. No. sc-29528) and 120 pmol of NRF-1 siRNA (Cat. No. sc-38105) or control siRNA-A (Santa, Cruz, Cat. No. sc-37007).

### Flow cytometry

Detection and quantification of *CD47* cell surface protein levels were performed using FACS and an anti-human *CD47* antibody (BioLegend, Cat. No. 323106). Briefly, cells were cultured in an exponential phase and harvested before reaching confluency. To harvest the cells, the spent medium was carefully aspirated, cells were washed with PBS, and detached using Accutase™ solution (STEMCELL, Cat. No.07922) for 3 min at 37°C in an incubator with 5% CO_2_. The cells were then collected and washed twice with PBS and resuspended in 100 μL of ice-cold cell staining buffer (CSB) (PBS, 1%BSA) at a concentration of 0.5-1x10^6^cells/ml. Following resuspension, cells were stained with anti-human *CD47*-FITC antibody (1μg/1x10^6^cells) and incubated on ice protected from light for 20 min. After incubation, the cells were centrifuged at 200g for 5 min, the supernatant was carefully aspirated, and the cells were washed twice with 200 μL of CSB. The cells were transferred to FACS tubes and analyzed in a BD FACSymphony A5 cell analyzer, at least 1x10^4^ cells live cells were recorded per cell type. Dead cells were excluded with a DAPI staining solution (10μg/mL). Once flow cytometry analysis was completed, data was analyzed in FlowJo v10.10 software, live cells were gated, and the geometric mean fluorescence intensity (GMFI) was obtained for each cell type. GMFI values were normalized (same number of live events per cell type) and compared between cell lines as well as their respective histograms.

### qRT-PCR

Cells were grown *in-vitro* on p100 cell culture dishes until reaching ~70% confluency after which they were lysed using 1ml of Trizole reagent. mRNA was precipitated using isopropanol and washed twice with 70% ethanol. cDNA was synthesized using a high-capacity cDNA reverse transcription kit (4368814) and power SYBR green PCR MasterMix (4367659 Thermo Fisher Scientific) was used to quantify total levels of *CD47* or NRF-1 transcripts; 18s gene was used for the normalization of qPCR results. Primer pair sequences are listed in **Supplementary Table 1**.

### ATAC-sequencing

For ATAC-Seq 50,000 cells were collected, washed in 50 μl of cold PBS, and resuspended in 50 μl of cold lysis buffer. Nuclei were isolated by centrifugation at 500 x g for 10 min at 4°C and then incubated with a transposition reaction mix for 30 min at 37°C. Following transposition, DNA was then purified using the MinElute PCR Purification kit (Qiagen, Hilden, Germany). The transposed DNA fragments were barcoded initially amplified for 5 cycles, and further amplified by standardized PCR. The resulting ATAC libraries were sequenced on an Illumina HiSeq 2500 platform in Rapid Mode with the 50-bp paired-end reads output mode. Raw reads were aligned to the human genome reference (1000 Genomes) using BWA-MEM (version 0.7.5a) with default settings. ATAC-Seq peaks were identified using the callpeaks function in MACS2 with a threshold set to -q = 0.01. The resulting peak calls were filtered for sequences that mapped to the mitochondria using shell scripts. BigWig files were normalized and integrated with publicly available genomic and epigenomic data for visualization in the UCSC Genome Browser.

### ChIP assay

For the ChIP assay cells were prepared using truChIP Chromatin shearing kit (PN520127) from covaries. Briefly, cells were cultured in 2 x p150 dishes and treated with fixing buffer have 1% formaldehyde for 10 mins followed by quenching of crosslinking with a quenching buffer for 5 mins. Cells were lysed per the manufacturer’s protocol and isolated nuclei were transferred into AFA tubes (Cat# 520135 covaris) and chromatin shearing was performed for 15 mins in E220 evolution ultrasonicator from covaries with recommended settings. 50μl of sheared chromatin sample was used to isolate DNA (**Supplementary Figure S2**) using a DNA purification kit from cell signaling technologies (cat# 14209S) thus the total amount of DNA in sheared chromatin was calculated using nanodrop. For immunoprecipitation, the buffers (cat# 14231) and ChIP-grade magnetic beads (cat# 9006S) were purchased from cell signaling and used as per the manufacturer’s protocol. Briefly, 5-7μg of DNA was used per reaction and 2% input material was set aside for quantification using qPCR. Each reaction was incubated overnight with 2.5-3μg of NRF-1, or isotype control IgG antibody followed by 2 hours of incubation with magnetic beads the following day. Immunoprecipitation was performed using a magnetic rack. After successful elution of pulled-down chromatin, DNA was isolated, and real-time qPCR was performed using power SYBR green PCR master mix (cat# 4367659) from Thermo Fisher Scientific. Primer pair sequences are listed in **Supplementary Table 1**.

### Generation of stable cell lines expressing *CD47* promoter reporters

*CD47* promoter reporters containing various numbers of NRF-1 binding sites were created by synthesizing respective DNA regions and cloning them into pGF1-4eCOL2A1_LUC lentiviral vector using BamHI and Spe1 restriction sites. Viral packaging was done in HEK293 cells and collected virions were used to infect target cells for two consecutive days. The *CD47* promoter activity of endogenous NRF-1 was assayed by measuring Luciferase bioluminescence.

### Datasets Used for *In-Silico* Analysis

TCGA TARGET GTEx study was used to analyze *CD47* expression levels in human melanoma tissues. ENCFF323SMV, ENCFF862EDR, ENCFF605IET, ENCFF785WIA, ENCFF000XJF, ENCFF644ITQ from encodeproject.org studies were used to analyze NRF-1 ChIP seq data across different types of cancer. Patient-derived melanoma cell lines were analyzed for chromatin accessibility at the *CD47* promoter region using the data from the study GSE134432. The ATAC-Seq data for the melanoma patient tissues was fetched from https://gdc.cancer.gov/about-data/publications/ATACseq-AWG. For *in-silico* H3K4^Me3^ analysis of patient-derived melanoma tissue from the database GSE33930, bowtie aligner was used to align the sequence reads, followed by the emoval of mitochondrial reads and duplicates using samtools. Macs2 was used to invoke callpeaks function to obtain peakfiles which were used to generate bigwig datafiles to be visualized in IGV.

## Results

### *CD47* upregulation in melanoma occurs at the mRNA level and is mediated by an open chromatin at its DNA promoter region

**Figure 1:**
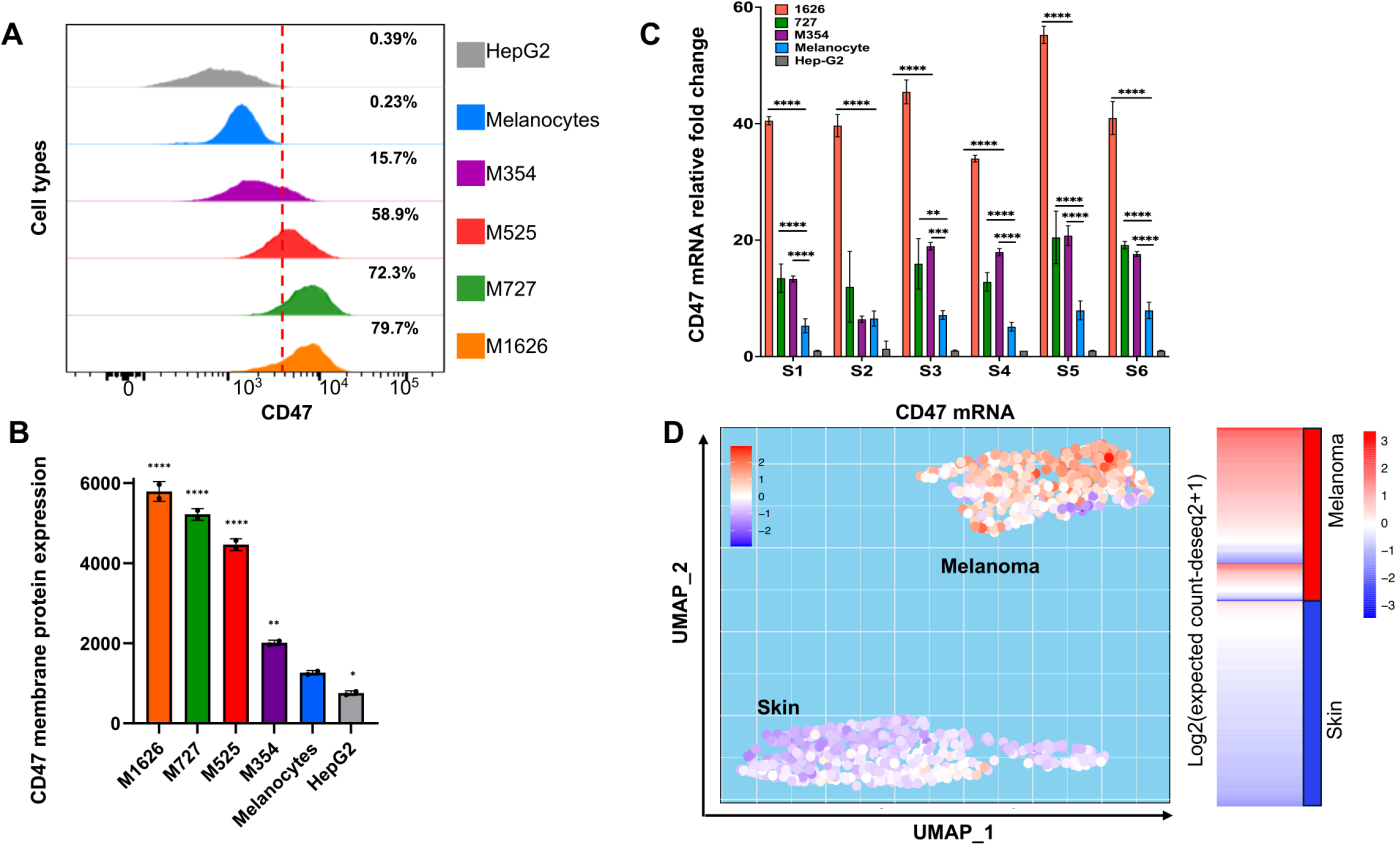
Upregulation of *CD47* expression during melanomagenesis. **(A)** Flow cytometry analysis of *CD47* expression in indicated cells. **(B)** Expression of *CD47* protein on the membrane of indicated cells. *CD47* expression is represented as normalized geometric mean fluorescent intensity (GMFI). **(C)** Relative expression of *CD47* mRNA in fold changes comprehensively evaluated by six primer sets to capture all the *CD47* mRNA isoforms abundance levels. **(D)** Unsupervised dimensional reduction analysis of a dataset from TCGA TARGET GTEx study was performed to visualize the distinctive clusters and expression of *CD47* in melanoma and normal skin samples. The transcript matrix was processed in R studio package called “umap” for UMAP generation. Samples with higher *CD47* in melanoma as seen on the heatmap represent the “Melanoma cluster”, and samples with lower *CD47* expression represent the “Skin” cluster in UMAP.

We previously established that high levels of *CD47* protein on freshly isolated human tumor cells are associated with melanoma progression and immune evasion (2). To understand whether augmented *CD47* expression results from an increase in corresponding mRNA production, we used metastatic melanoma patient-derived cells M727, M354, and M1626 that were determined to express various levels of *CD47* (**Figures 1A, B**) and compared them to normal melanocytes or hepatocellular carcinoma cells, HEPG2, (known for low levels of *CD47* protein) using qRT-PCR. *CD47* gene is comprised of 11 exons and as a result of splicing, most notably between exons 8-10, multiple mRNA isoforms are translated (**Supplementary Figure 1A**). Therefore, to prospectively quantify levels of all *CD47* transcript isoforms we designed multiple primer sets for a common exon 2, as well as, two primer sets that amplify mRNA regions between exon 2-4 and exon 7-8 (**Supplementary Figure 1B**, **Supplementary Table 1**). After performing multiple qRT-PCR assays, our results demonstrate that all metastatic melanomas expressed 10-15 folds (depending on the isoform) elevation in *CD47* mRNA as compared to normal melanocytes or HepG2 cells (**Figure 1C**). To further explore clinical significance of our findings, we investigated *CD47* mRNA levels among 422 melanoma subjects in comparison to 123 normal skin subjects using TCGA TARGET GTEx dataset. Utilizing an unsupervised dimensional reduction approach, we generated UMAP graphs that visualized patterns of clusters across diagnosed subjects and levels of *CD47* mRNA expression. Significantly, two distinct clusters were formed based on the *CD47* mRNA levels, high and low, which corresponded to the malignant melanoma patients cluster (high) and the normal skin cluster (low) (**Figure 1D**).

**Figure 2:**
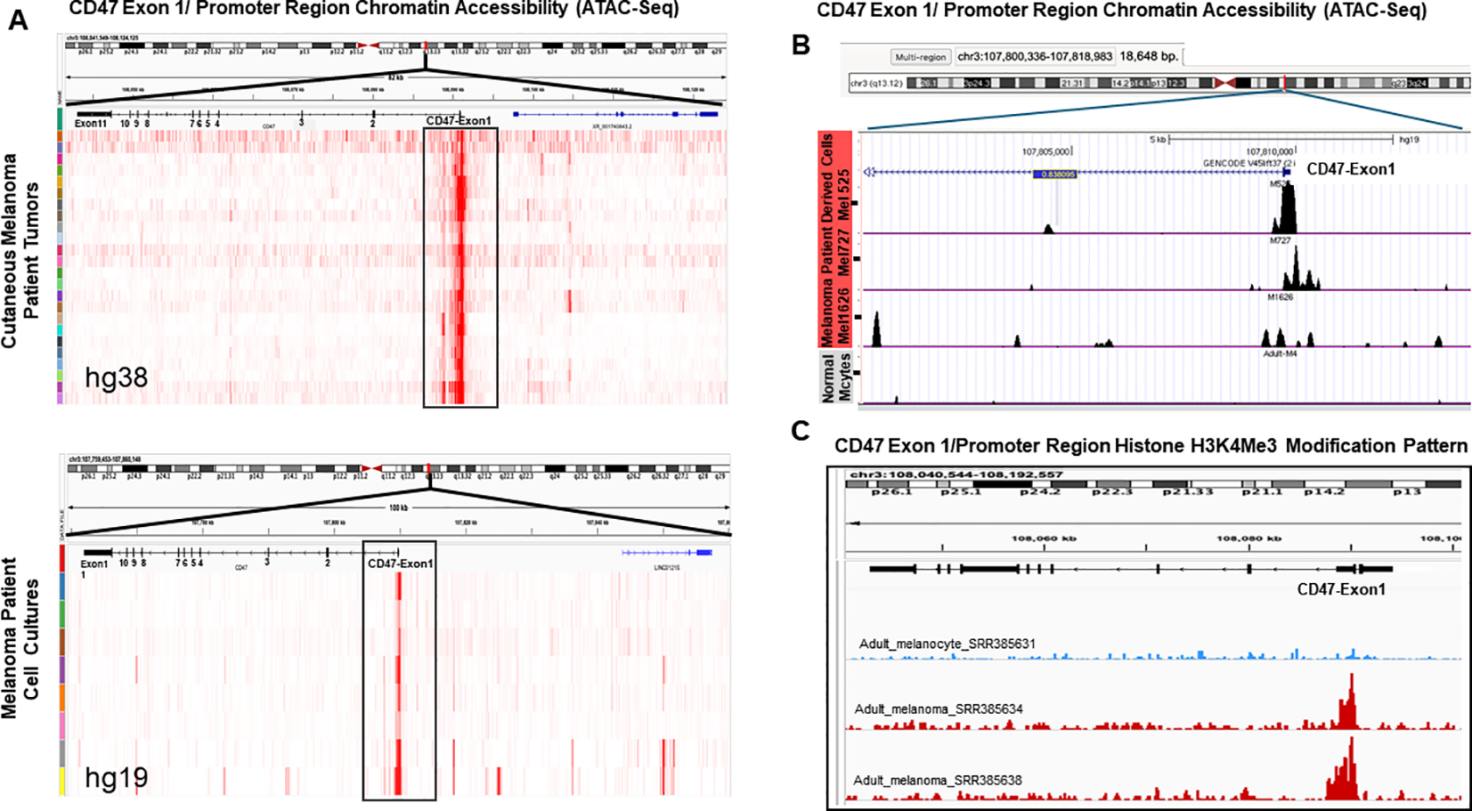
Changes in chromatin landscape of *CD47* promoter region in patient melanomas and patient-derived melanoma cell lines. **(A)** Analysis of ATAC-seq peak signals around the *CD47* DNA promoter region in surgically removed melanoma patient tumors and established cell lines using SKCM and GSE134432 databases. **(B)** Analysis of *CD47* promoter region chromatin structure using ATAC-Seq approach on independent patient-derived melanoma cell lines (M727, M1626, M525) and normal melanocytes. **(C)** Analysis of H3K4^Me3^ histone modification mark at the *CD47* promoter DNA region using melanoma patient samples and melanocytes from the database GSE33930. Peaks were called using Macs2 and peak files in the Bigwig format were visualized in the IGV 2.7 version.

To understand molecular mechanisms of augmented *CD47* mRNA expression in malignant melanoma, we investigated the dynamics of chromatin re-modeling at *CD47* promoter using ATAC-Seq. This approach allows the generation of a cell-specific chromatin accessibility landscape to determine the level of open chromatin and subsequent promoter activation. First, we used *in-silico* ATAC-Seq analysis of the skin cancer melanoma (SKCM) dataset (https://gdc.cancer.gov/about-data/publications/ATACseq-AWG) containing malignant tissues obtained from 13 melanoma subjects (11 samples were analyzed in duplicates) and a melanoma dataset (GSE134432) containing 9 genetically homogenous melanoma patient-derived cell lines to gain insights into the accessibility of the *CD47* promoter region. Our results demonstrate that in both freshly resected melanoma samples, as well as, in melanoma patient-derived cell lines, the DNA region corresponding to the *CD47* promoter has a significantly increased chromatin accessibility which could be conjectured as a sign of active mRNA transcription (**Figure 2A**). To validate these changes in the chromatin landscape of the *CD47* promoter region during melanomagenesis, we performed ATAC-seq on multiple independent patient-derived melanoma cell lines in comparison to normal adult melanocytes. Importantly, we were able to detect strong peak signals associated with an open chromatin structure around the promoter of *CD47* in malignant melanomas, which were missing in normal adult melanocytes indicating the closed chromatin structure of the *CD47* DNA promoter region in non-transformed cells (**Figure 2B**).

Open chromatin status is often associated with specific histone modifications such as H3K4^Me3^, which is commonly found at the promoter regions of actively transcribed genes (20). To determine whether this mechanism can account for an increased *CD47* mRNA production during melanomagenesis, we performed *in-silico* analysis of H3K4^Me3^ histone modifications at the *CD47* promoter in melanoma patients and normal adult melanocytes using the GSE33930 dataset. Our results demonstrate that peak signals for H3K4^Me3^ DNA modification at the *CD47* promoter region are significantly stronger in malignant melanomas in comparison to normal melanocytes (**Figure 2C**).

### Nuclear respiratory factor-1 TF binds to the *CD47* promoter with preferential affinity to the most proximal region in melanoma

**Figure 3:**
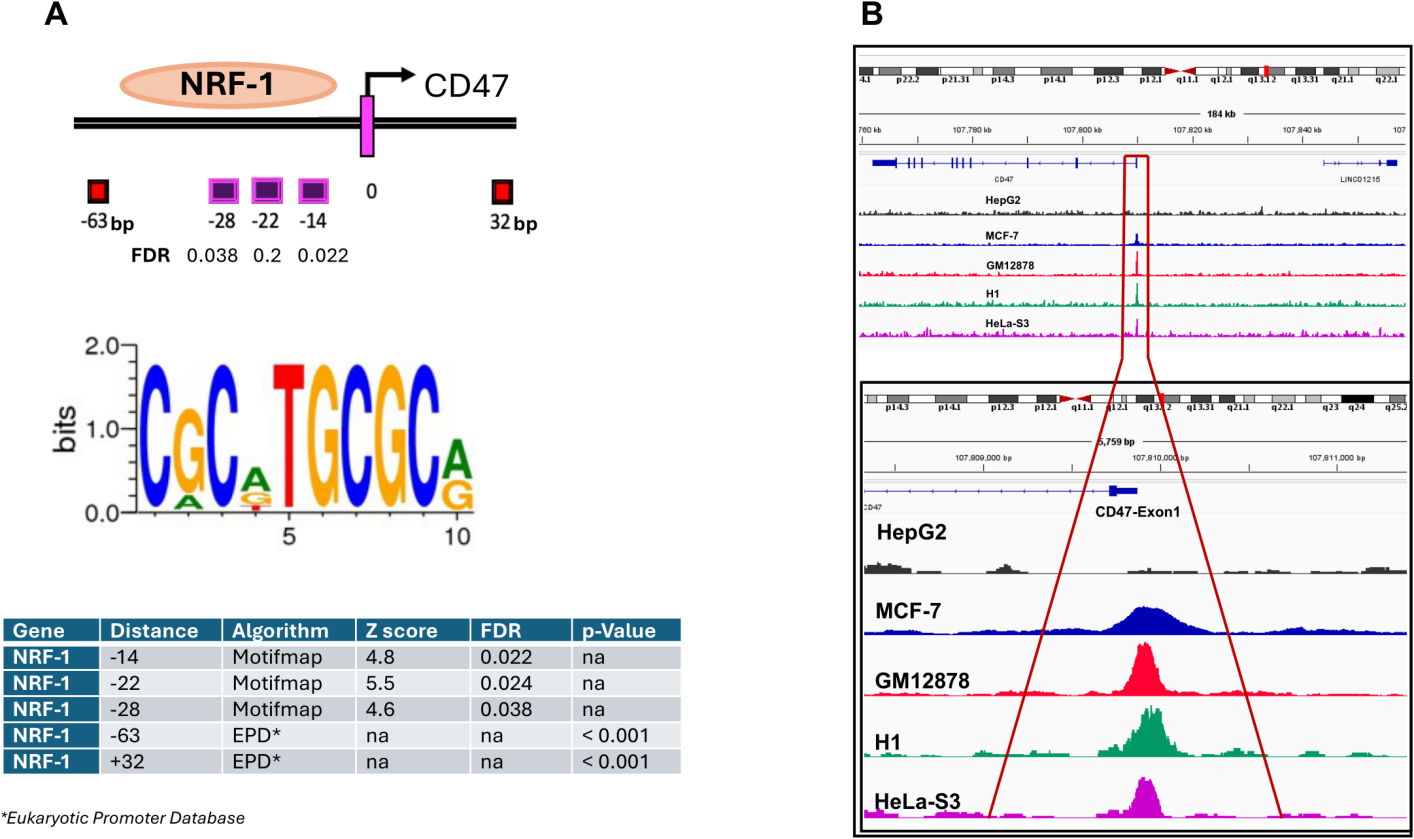
Identification of NRF-1 TF as the regulator of *CD47* mRNA expression in melanoma. **(A)** Numerous NRF-1 TF binding sites were predicted and identified in the proximal region of the *CD47* Exon1 DNA Region. The consensus sequence required for the NRF-1 TF binding was extracted using the Motifmap algorithm. **(B)** Analysis of publically available ChIP-seq datasets for indicated cancer types reveals NRF-1 binding at the *CD47* Exon1 promoter region.

To identify transcriptional regulators of the *CD47* promoter we analyzed its DNA region (± 1000bp relative to the transcription start site (TSS)) for the presence of potential TF binding sites using MotifMap and the Eukaryotic Promoter Databases. Results ranking based on FDR (< 0.038) and p-value (< 0.001) scores revealed a number of DNA binding sites within that region (-63bp, -28bp, -22bp, -14bp, and +32bp), all corresponding to the NRF-1 consensus sequence (**Figure 3A**). To validate predicted interactions of NRF-1 with *CD47* promoter we first performed *in-silico* analysis of ChIP-seq datasets derived from different types of cancer. Importantly, strong peak signals corresponding to NRF-1 binding at the *CD47* promoter were identified in the majority of tumors (results presented as fold changes over control), except for HepG2 hepatocarcinoma (**Figure 3B**), which was found to express low *CD47* protein and mRNA transcript levels (**Figure 1**).

**Figure 4:**
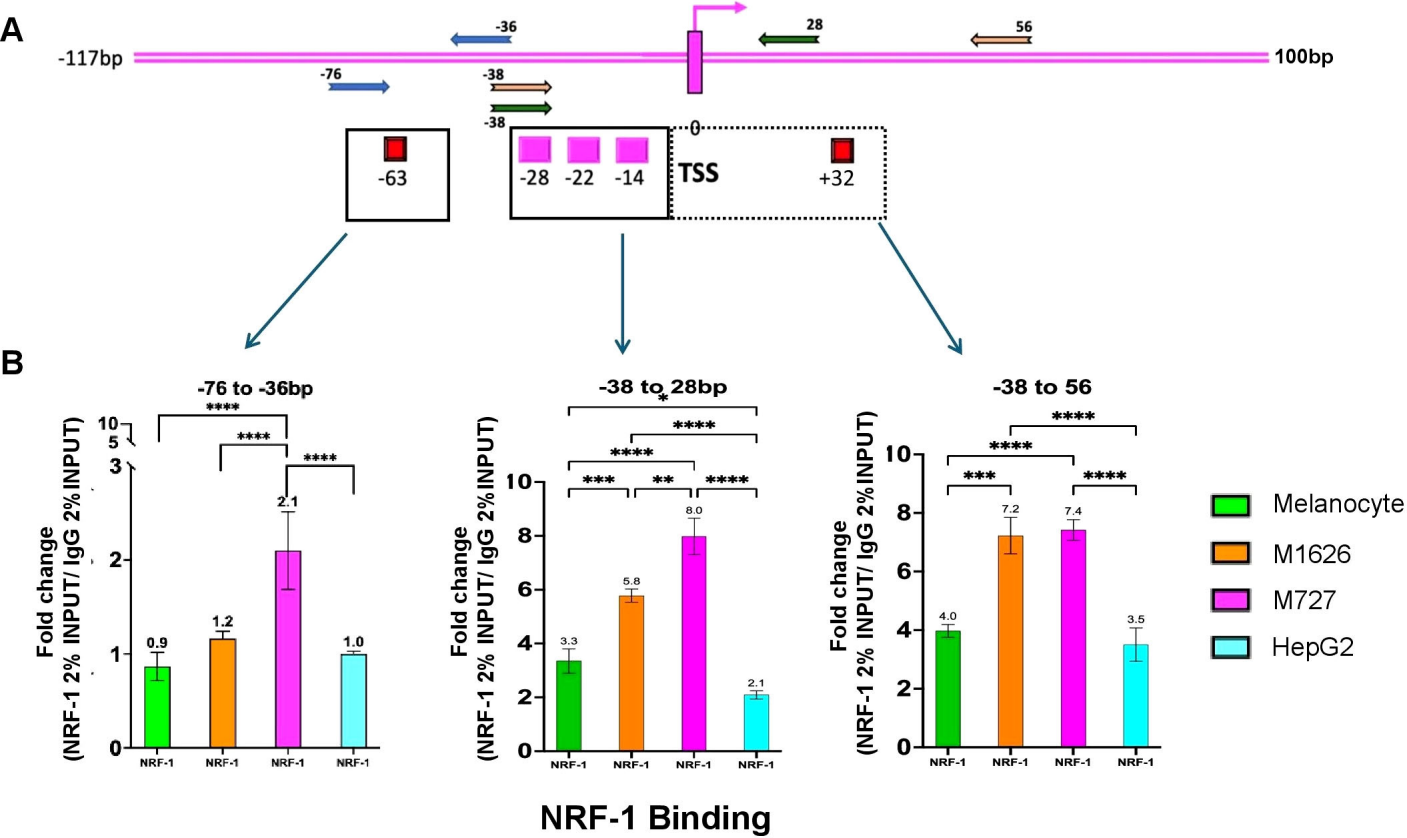
NRF-1 TF differentially binds at the proximal *CD47* promoter region spanning between -38 to +56bp relative to TSS. **(A)** Various sets of primers to target and enrich indicated regions of predicted NRF-1 binding at *CD47* promoter. **(B)** ChIP assays demonstrating differential affinity binding of NRF-1 at predicted DNA regions of *CD47* promoter. The strongest affinity for melanoma cells was determined for binding sites located between the -38 to 56bp region of the *CD47* promoter.

Next, to determine NRF-1 regulation of *CD47* promoter, we performed Chromatin Immunoprecipitation (ChIP) assays on malignant patient-derived melanoma cells, as well as, adult normal melanocytes and HepG2 cells. Following incubation of the sheared chromatin (500-1000bp) with NRF-1 Ab the pulled-down DNA fragments were quantified by PCR using primer sets designed to amplify only the *CD47* promoter regions spanning different putative NRF-1 binding sites (**Figure 4A**). To precisely delineate NRF-1 DNA binding regions within the *CD47* promoter, we designed multiple primer pairs to enrich for the following sites relative to TSS: Set 1 (-38/+28) includes putative binding sites at -28, -22, and -14bp; Set 2, (-38/+56) includes putative binding sites -28, -22, -14, and +32; Set 3 (-63/-36) includes putative binding site at position -63bp only (**Figure 4A**, **Supplementary Table 1**). An additional set of primer spanning the promoter region of MEF2A gene (a validated NRF-1 binding target) was used as a positive control for all ChIP experiments (**Supplementary Figure 2**). ChIP analysis on the above indicated cell types identified NRF-1 loading at the predicted DNA region of the *CD47* promoter with differential affinity for proximal and distal binding sites (**Figure 4B**). Thus, in malignant melanomas there was a significant increase in binding affinity at the most proximal sites (-28, -22, -14) as compared to normal melanocytes (1.7-fold for M727 and 2.4-fold for M1626). Moreover, when compared to HepG2 cells, which have been characterized by the lowest levels of *CD47* mRNA and protein, differences in NRF-1 binding affinity were found to be even greater (1.85-fold for M727 and 3.8-fold for M1626 **Figure 4B** middle panel). Inclusion of the proximal downstream binding site (+32bp) resulted in similar differences in the NRF-1 binding affinity between malignant melanoma and low *CD47* expressing cells (adult melanocytes and HepG2 cells) (**Figure 4B** right panel). However, differences in NRF-1 binding affinity were diminished at the more distal binding site (-63bp) within the *CD47* promoter region (**Figure 4B** left panel). In summary, these findings indicate that *CD47* transcriptional regulation by NRF-1 is determined by the proximal promoter region in melanoma cells.

### Effective activation of *CD47* promoter requires multiple NRF-1 binding sites between -120bp to +50bp promoter region

**Figure 5:**
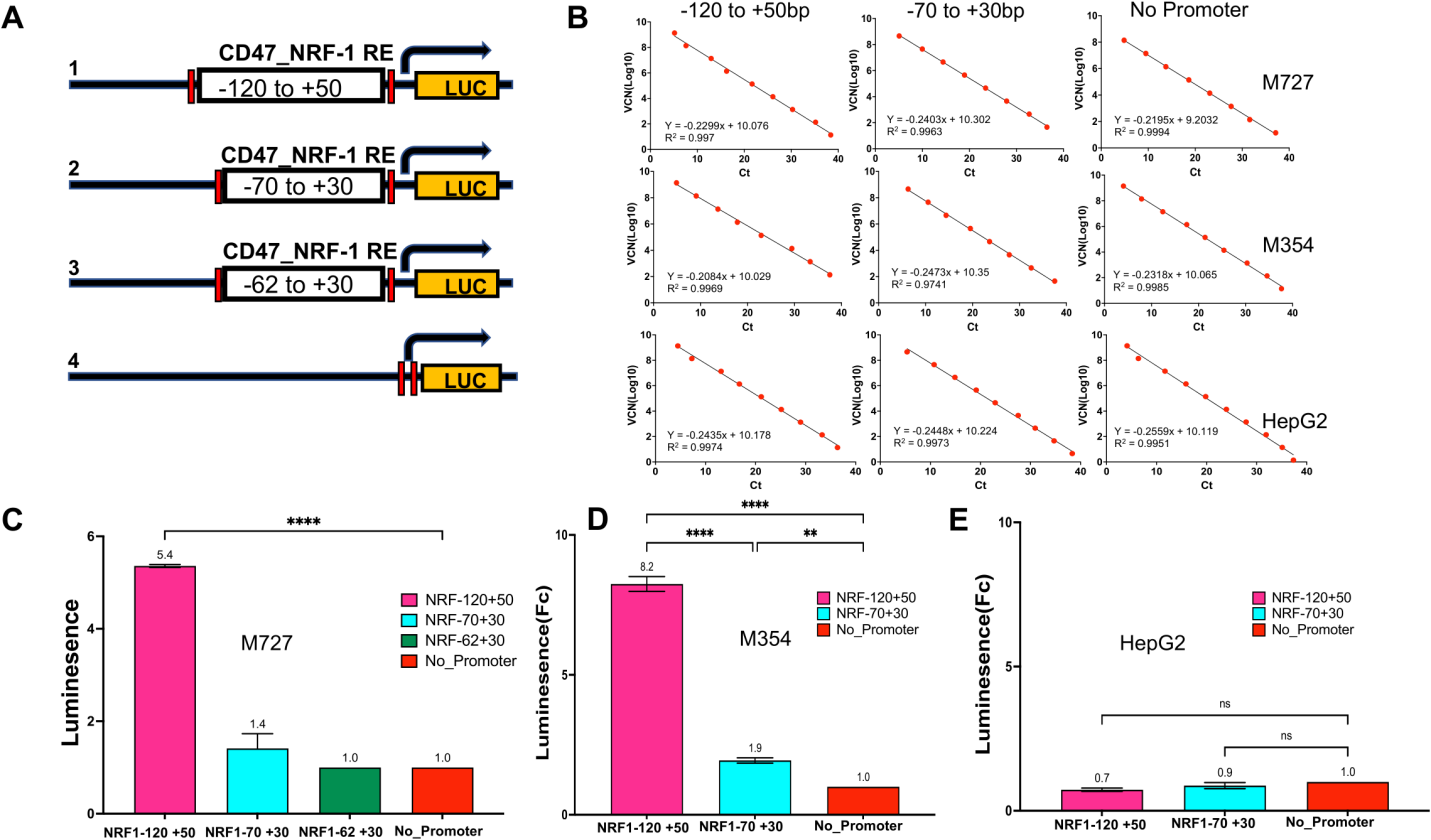
Activation of *CD47* promoter requires DNA region containing multiple NRF-1 binding sites located between -120bp to +50bp relative to TSS. **(A)** A series of Luciferase reporter constructs harboring different lengths of *CD47* promoter region were generated to assay the NRF-1 mediated *CD47* promoter activation. **(B)** Luciferase signals were read using a 96-well plate reader and normalized to viral copy number (VCN) and total protein. VCNs were calculated for each plasmid construct for every cell line separately using serial dilutions of plasmids to generate a linear regression curve with a slope equation that was further used to calculate the VCN of samples. **(C–E)** Relative CD47_NRF1 Luc reporter assay signal in fold changes for M727, M354 and HepG2 cells. A construct with no CD47 promoter region was used as a negative control.


Initiation of transcription from eukaryotic promoters often requires binding of the TFs at numerous locations within the promoter region. Identification and validation of multiple NRF-1 TF binding sites within the proximal region to the *CD47* TSS (200 bp window) prompted us to investigate the minimum adequate length of DNA required for the *CD47* promoter activation. To achieve this goal, we designed and cloned a series of bioluminescent reporter plasmids expressing Luciferase gene under the control of the proximal *CD47* promoter region containing various numbers of NRF-1 TF binding sites (**Figure 5A**). Specifically, construct 1 contained all five NRF-1 proximal binding sites and covered the DNA region from -120 to +50bp relative to the TSS, construct 2 contained four out of five NRF-1 binding sites and covered the region from -70 to +30 bp, and lastly construct 3 contained only three out of five NRF-1 binding sites and covered the region from -62 to +30bp of *CD47* promoter relative to the TSS. Lastly, we also used a complete promoter deletion variant of Luciferase expressing plasmid as a negative control (construct 4 **Figure 5A**). All of the above variants of the *CD47* promoter region were synthesized and cloned into the lentiviral plasmid pGF1-4eCOL2A1_Luc (21) using BamHI and Spe1 restriction sites. In order to evaluate endogenous NRF-1 activity at the *CD47* promoter, we generated melanoma cell lines to stably express all versions of Luc reporters using previously published lentiviral transduction protocols (22, 23). To obtain statistically significant results, raw Luc bioluminescence values were first normalized to the viral copy numbers (VCN) for each reporter plasmid. Briefly, serial dilutions of the known concentration of each Luc reporter plasmid were premixed with 5ng of carrier genomic DNA (isolated from each of the above-indicated cell lines), and the real-time PCR was performed using Luciferase primers. Obtained CT values were used to generate a standard curve which allowed to calculate the VCN values for the known concentration of plasmids according to the given formula:

$$\begin{array}{c} Number\;of\;copies\;\left(=\;molecules\right)\\ \;=\;(X\;ng\;*\;6.02214\;x\;10^{23}molecules/mole)/\left(\left(MW\;g/mole\right)\;*\;1\;x\;100\;ng/g\right) \end{array}$$

$$\begin{array}{c} Where:X=amount\;of\;amplicon\left(ng\right);N\\ \;\;\;\;=length\;of\;dsDNA\;amplicon;MW\\ \;\;\;\;=molecular\;weight\;of\;plasmid \end{array}$$

Thus obtained linear regression curves allowed us to calculate viral copy numbers for an equal amount of genomic DNA for each individual cell line (**Figure 5B**). Moreover, raw bioluminescence values were also normalized to the amount of total protein present in each lysate. Normalized data demonstrated that initiation of *CD47* transcription requires the presence of all five NRF-1 DNA binding sites (**Figure 5C**). Thus, Luc activity was induced 5-8 fold in melanoma cells containing full-length proximal *CD47* promoter (Construct 1) as compared to low *CD47* expressing HepG2 cells (**Figure 5C**), while *CD47* promoter region containing a reduced number of NRF-1 binding elements (Constructs 2 and 3) had marginal or no effect on reporter activity in the same cells (**Figures 5C–E**). In conclusion, our data demonstrates that the promoter region required for efficient *CD47* transcriptional activation in melanoma lies between -120 to +50bp relative to the TSS.

### NRF-1 activity directly contributes to the CD47 expression in melanoma

**Figure 6:**
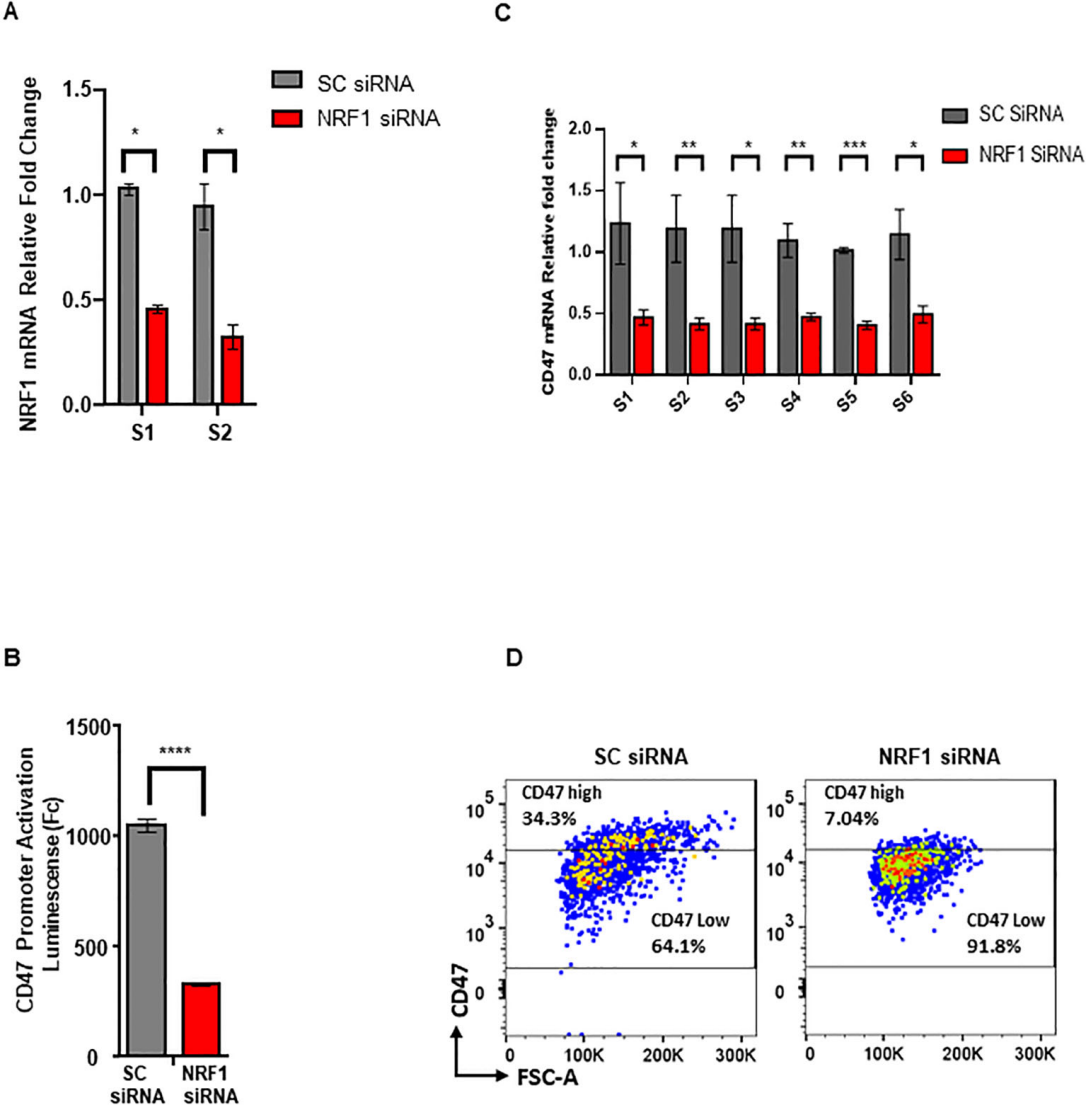
NRF-1 downregulation reduces expression of CD47 in melanoma. **(A)** Quantification of NRF1 mRNA following siRNA mediated knockdown in melanoma cells using qRT-PCR and two independent sets of primers, S1 and S2. **(B)** Quantification of CD47 prmoter activation using Luc reporter assay in the cells transduced with NRF1_siRNA or control, SC_siRNA. **(C)** Quantification of CD47 mRNA following siRNA mediated NRF1 knockdown in melanoma cells using qRT-PCR and six independent sets of primers, S1- S6. **(D)** Analysis of cell surface CD47 protein levels using FACS for cell populations transduced with NRF1_siRNA or control, SC_siRNA.

To demonstrate functional significance of NRF1 TF in the regulation of *CD47* we tranduced melanoma cells expressing *CD47*_Luc promoter reporter (Construct 1 described above) with siRNAs (a pool of 3) targeting NRF1 mRNA to achieve its downregulation (**Figure 6A**). Subsequent quantification of Luc signal revealed a significant reduction in *CD47* promoter activity (by more than 60% (p=0.0149)) in cells transduced with NRF1 siRNA as compared to matching controls transduced with SC siRNA (**Figure 6B**). Next, we profiled these cells for an endogenous *CD47* mRNA levels using qRT-PCR and multiple sets of primers corresponding to its various regions. Our data demonstrates that NRF1 downregulation causes at least 50% decrease in *CD47* mRNA expression in target cells (p=0.0245 - 0.00361) (**Figure 6C**). Lastly, we used FACS and fluorescent conjugated antibodies to quantify *CD47* protein on the cell membrane, critical aspect of its immune modulatory function during tumor progression. Our analysis demonstrates that NRF1 downregulation leads to the significantly reduced proportion of melanoma cells expressing high levels of this innate immunity checkpoint when compared to the original cell population trasduced with control SC-siRNA: 7.04% *CD47* ^high^//91.8% *CD47* ^low^ for NRF1-siRNA and 34.3% *CD47* ^hig^//64.1% *CD4*7^low^ for SC-RNAi transduced cells (**Figure 6D**). In summary, the above described results provide strong experimental evidence to indicate that NRF1 is directly involved in regulation of *CD47* expression in melanoma.

## Discussion

Cluster of Differentiation 47 (*CD47*), also known as Integrin Associated Protein (IAP), is a transmembrane protein belonging to the immunoglobulin superfamily (Ig) which regulates a variety of cell processes including, cell adhesion, motility and apoptosis (24–26). It gained considerable attention in the past decade after its function as a potent innate immunity checkpoint regulator was characterized first for hematopoietic malignancies and later for solid tumors, including melanoma (2, 27–29). Interaction of *CD47* with the signal regulatory protein-alpha (SIRPα) receptor expressed on many myeloid derived cell lineages activates molecular pathways effectively inhibiting an anti-tumor function of innate immunity (30). Upregulation of *CD47* by tumor cells in order to avoid immune recognition and elimination is thus regarded as one of the critical steps during carcinogenesis. Therefore, revealing mechanisms involved in the regulation of *CD47* expression represents a pivotal task in our understanding of tumor progression and immune evasion. Importantly, highly diverse mechanisms of malignant transformation and cancer progression can result in different modes of *CD47* regulation depending on the tissue type of tumor origin. For example, NFκB TF has been shown to activate *CD47* expression by binding to the distal enhancer elements in cells derived from breast, cervical, and non-small lung cell carcinomas (18, 19, 31). Other oncogenic TFs have also been reported to affect *CD47* expression directly as a part of an oncogene-driven process of malignant transformation. Thus, hypoxia inducible TF, HIF-1α, binds to the *CD47* promoter region at -239bp and -200bp relative to the TSS and induces its expression in response to hypoxic conditions *in-vitro* in MCF7 cells (17). Other studies implicated MYC, a well-characterized TF with strong oncogenic potential, in the transcriptional regulation of *CD47* during the progression of leukemia using MYC-induced T cell acute lymphoblastic leukemia mouse model; MYC T-ALL, and human leukemia cells lines; CCRF-CEM and Jurkat (16). Interestingly, binding sites for MYC TF were also identified in our analysis of *CD47* proximal promoter region (**Supplementary Figure 3A**), however, MYC binding was undetectable in human malignant melanoma within *CD47* DNA promoter region between -120bp to +54 relative to TSS required for its activation (**Supplementary Figure 3B**).

In our present work, we used numerous clinical human melanoma samples, as well as, previously collected pathological datasets of disease progression to establish that the tumor-specific upregulation of myeloid checkpoint molecule, *CD47*, occurs at the mRNA level during melanomagenesis. We reveal that it is mediated by a significant increase in chromatin accessibility at *CD47* prmoter region, including elevated levels of epigenetic histone modifications at H3K4^Me3^ residues, in melanoma but not in normal melanocytes or HepG2 cells which are known for low *CD47* expression. Using serial DNA deletion analysis in combination with bioluminescence reporter systems, we determine that the minimum promoter region required for *CD47* transcriptional activation in melanoma lies between -120 to +50bp relative to the TSS. Furthermore, we identified NRF-1-TF as a specific transcriptional regulator of *CD47* promoter, which was found to be physically associated with multiple binding sites at the promoter region using ChIP approaches. Our data demonstrate that NRF-1 binding to the most proximal sites at -28, -22, and -14bp sites relative to the TSS appears to be a critical step during *CD47* promoter activation which distinguishes it from the cells of low *CD47* levels. This indicates that the basal level of *CD47* transcription can be initiated by NRF-1 binding to the distal regions of the *CD47* promoter starting at -63bp (which is detected for all cell types); however, upregulation in *CD47* mRNA synthesis occurs only in response to NRF-1 occupying additional proximal sities indicated above. Importantly, downregulation of NRF1 causes notable reduction of *CD47* mRNA (>50%) and as a result, significant decrease in melanoma cell poolations with high *CD47* levels. At the same time *CD47* expression was not completely abolished in response to NRF1 downregulation. pointing to the fact that other TFs mentioned above (such as NFkB and HIF1a) whose biding sites are identified within *CD47* promoter/enchancer regions can contribute to its expression. Further studies focused on combinatorial approaches to simultaneously modulate activity of NRF1 along with other TFs mentioned above at the *CD47* prmoter/enchancer DNA regions will be required to precisely delineate each TF contribution to the activity of *CD47* promoter overall.

Under normal conditions, NRF-1 TF plays a vital role in maintaining mitochondrial biogenesis, including oxidative phosphorylation, by transcribing multiple proteins involved in processes regulating biogenesis such as TFB1M, TFB2M, TFAM, and *cytochrome c* (32, 33). However, NRF-1 has also been implicated in the process of malignant transformation based on its role in energy metabolism. Specifically, studies using human hepatocellular carcinoma and colorectal tumor tissues show the significance of upregulated NRF-1 protein levels (mediated by neutrophil extracellular trap formation) in inducing mitochondrial biogenesis promoting tumor growth and metastasis (34). In addition, NRF-1 activity has also been associated with estrogen-induced breast carcinogenesis, and renal cell carcinoma where it regulates the expression of the *TFE3* gene required for cellular energy metabolism during proliferation (35, 36).

In our present study, we characterized a novel role of NRF-1 in melanomagenesis as a regulator of the major innate immunity checkpoint, *CD47*, in tumor cells. Importantly, binding of Pal/NRF-1 to the CD47 promoter was also previously reported in human neuroblastoma and hepatoma cell lines (37), as well as, during arising resistance to the oncogenic B-RAF molecular inhibitors (38). These findings open an opportunity for the development of modulatory compunds against NRF1 and functional testing of this TF involvment in an immune evasion by tumor cells during cancer progression. Clinical significance of these approaches can further be tested both *in-vitro* and *in-vivo* in combination with already established immunotherapeutic agents, such as PD1/PDL1/CTLA4 blocking antibodies, commonly used for the treatment of metastatic disease. Malignant transformation is a complex and multifaceted process that combines changes in both metabolism and immune recognition within cancer cells that successfully evolve to give rise to the more aggressive and metastatic tumors. NRF-1 TF therefore represents a key nodal point in this process due to its unique ability to transcriptionally regulate genes underlying both mitochondrial biogenesis and evasion of an innate immunity.

## Data Availability

Publicly available datasets were analyzed in this study. This data can be found here: ENCFF323SMV, ENCFF862EDR, ENCFF605IET, ENCFF785WIA, ENCFF000XJF, ENCFF644ITQ from encodeproject.org. GSE33930, GSE134432 from gene expression omnibus.

## Supplementary material

Supplementary Figure 1 Primer design for *CD47* real-time qPCR assay. **(A)** Schematic representation of different isoforms for the human *CD47* mRNA. **(B)** primer sets (6) that were used to quantify an abundance of indicated *CD47* mRNA isoforms.

Supplementary Figure 2 MEF2A gene promoter used as a positive control of anti-NRF-1 ChIP assays.

Supplementary Figure 3 Evaluation of MYC binding at the proximal *CD47* promoter region in melanoma. **(A)** MYC binding sites on the *CD47* region as predicted by Eukaryotic Promoter Database. **(B, C)** MYC ChIP on *CD47* promoter in melanoma cells M1626 and hepatocarcinoma cells HepG2. **(D)** MYC ChIP on Nmp-1 promoter (positive control) in M1626 and HepG2 cells.
