# Supplementary material for: NRF-1 transcription factor regulates expression of an innate immunity checkpoint, CD47, during melanomagenesis
Source: Front Immunol. 2024 Dec 17;15:1495032. doi: 10.3389/fimmu.2024.1495032 (PMC11685207; doi:10.3389/fimmu.2024.1495032)
Supplement: Supplementary file 1 [file Table1.docx]

|  | **ChIP** |  |
| --- | --- | --- |
|  | Primer | Position from TSS |
| FWD | GCGCGTGCGCGGCTCTCGC | -76 |
| REV | GCCCGCTCCCCCGCCTGC | -36 |
| FWD | GCGGGAAGCAGTGGGAGC | -38 |
| REV | TTACAGGCAGGACCGACCGC | 56 |
| FWD | GCGGGAAGCAGTGGGAGC | -38 |
| REV | GTCACAGGCAGGACCCA | 28 |
|  | **MEF2A (Control)** |  |
| FWD | ACCGAGAGGATAATTCAGTCCTG |  |
| REV | ACATCCGCGCACGGATC |  |
|  |  |  |
|  | **CD47 mRNA** | Primer pair Set # |
|  |  |  |
| FWD | GTGGGGACAGTGGACTTGTT | S1 |
| REV | TGGAGGCACAAAACACTACTGA | S1 |
| FWD | TCAGTGGGGACAGTGGACTT | S2 |
| REV | ATGGAGGCACAAAACACTACTGA | S2 |
| FWD | TGAGACAGCATCACTCTTATCCA | S3 |
| REV | AAGTCCACTGTCCCCACTGA | S3 |
| FWD | TGAGACAGCATCACTCTTATCCAT | S4 |
| REV | AGTCCACTGTCCCCACTGAC | S4 |
| FWD | AGAAGGTGAAACGATCATCGAGC | S5 |
| REV | CTCATCCATACCACCGGATCT | S5 |
| FWD | CATGGCCCTCTTCTGATTTC | S6 |
| REV | GGAGGTTGTATAGTCTTCTGATTGG | S6 |
|  |  |  |
|  | **NRF-1 mRNA** |  |
| FWD | GTACAAGAGCATGATCCTGGA |  |
| REV | GCTCTTCTGTGCGGACATC |  |
| FWD | GGAACACGGAGTGACCCAAA |  |
| REV | AGGCGAGTCTTCATCAGCAC |  |
|  |  |  |
|  | **Luciferase reporter** |  |
|  |  |  |
| FWD | GTGTTGGGCGCGTTATTTATC |  |
| REV | AGACGACTCGAAATCCACATATC |  |
|  |  |  |
