# Supplementary figures and images for: NRF-1 transcription factor regulates expression of an innate immunity checkpoint, CD47, during melanomagenesis
Source: Front Immunol. 2024 Dec 17;15:1495032. doi: 10.3389/fimmu.2024.1495032 (PMC11685207; doi:10.3389/fimmu.2024.1495032)

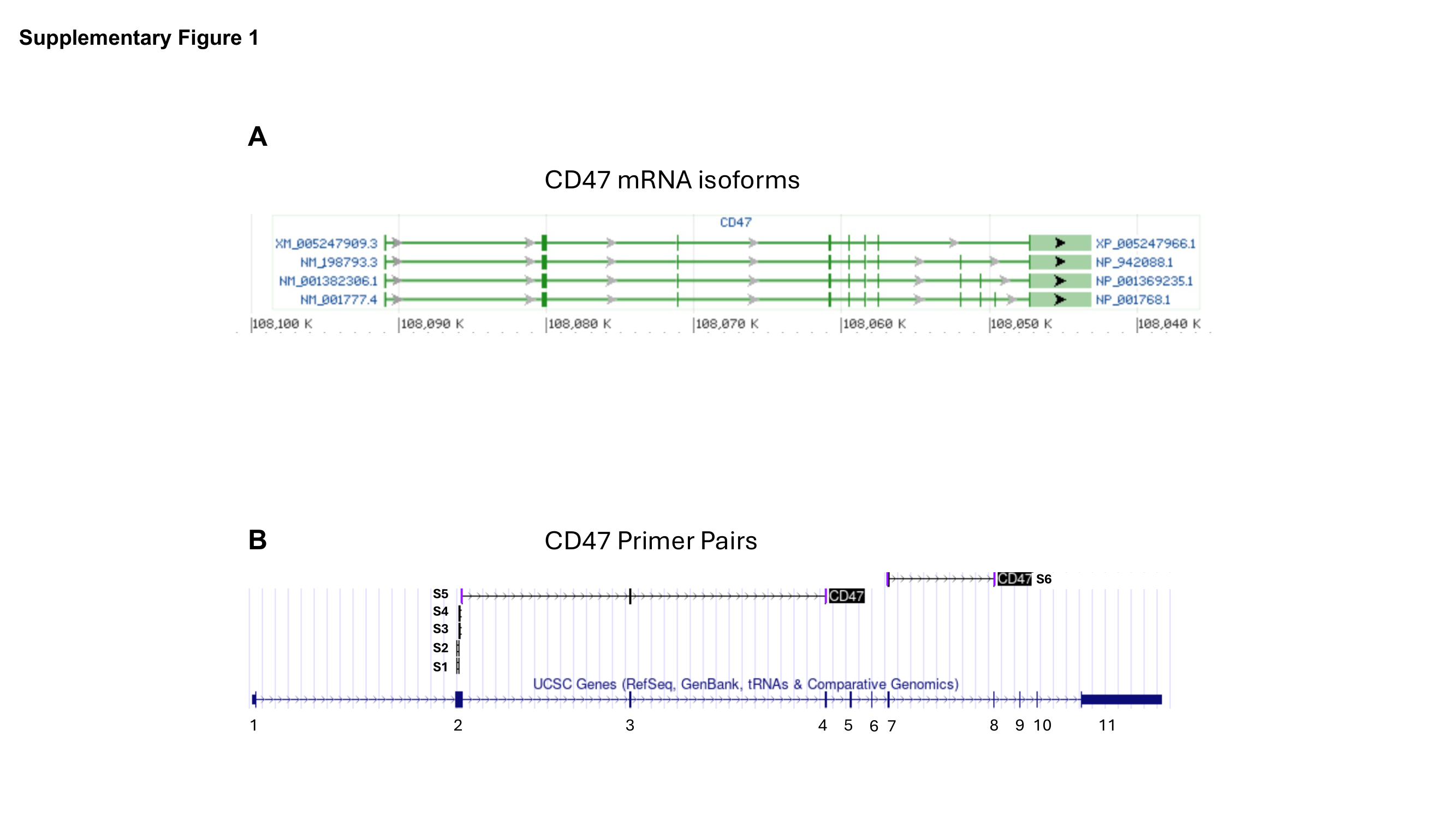

Supplement: Supplementary Figure 1 — Primer design for CD47 real-time qPCR assay. (A) Schematic representation of different isoforms for the human CD47 mRNA. (B) primer sets (6) that were used to quantify an abundance of indicated CD47 mRNA isoforms. [file Image1.tiff]

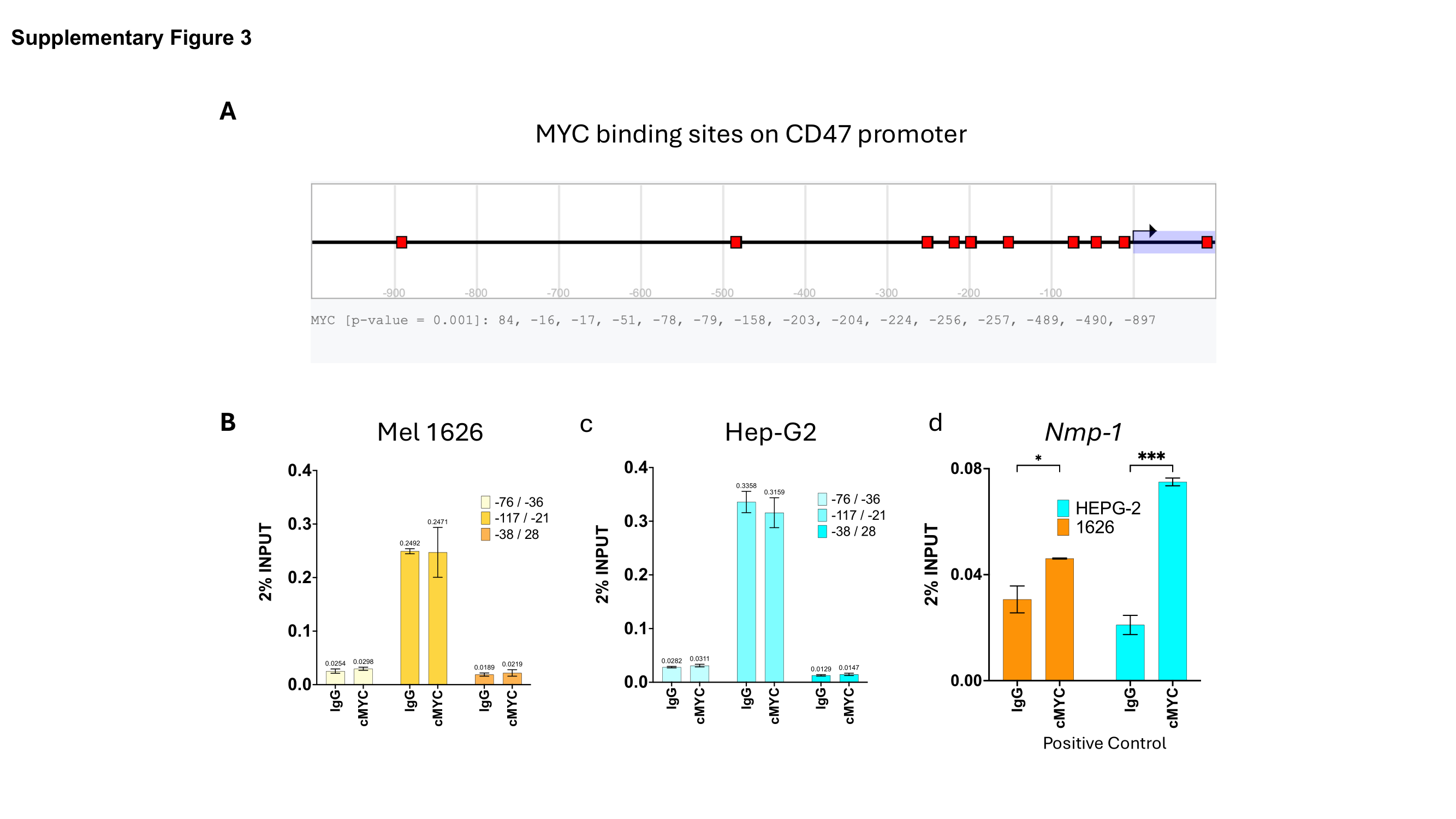

Supplement: Supplementary Figure 2 — MEF2A gene promoter used as a positive control of anti-NRF-1 ChIP assays. [file Image2.tiff]

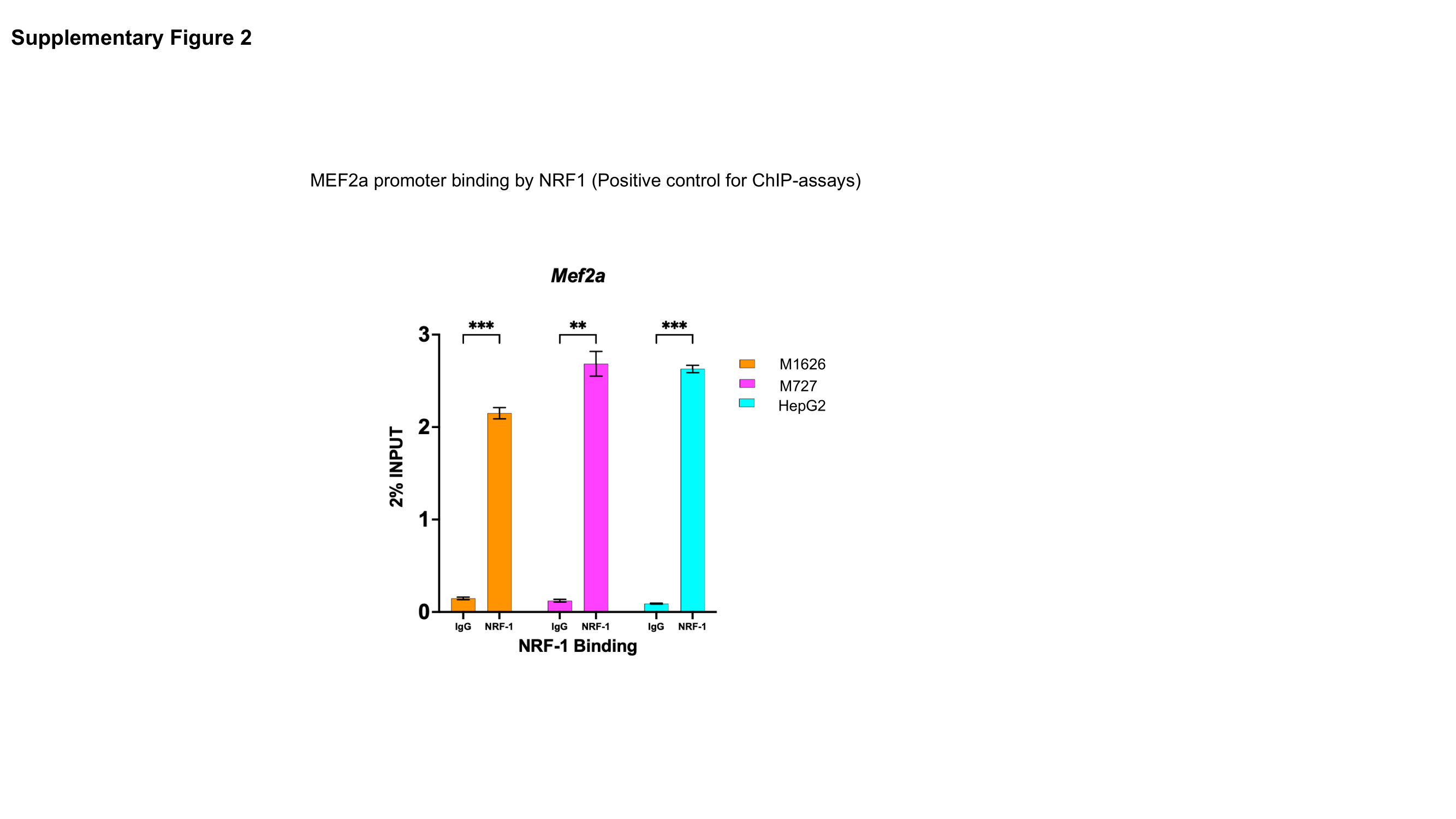

Supplement: Supplementary Figure 3 — Evaluation of MYC binding at the proximal CD47 promoter region in melanoma. (A) MYC binding sites on the CD47 region as predicted by Eukaryotic Promoter Database. (B, C) MYC ChIP on CD47 promoter in melanoma cells M1626 and hepatocarcinoma cells HepG2. (D) MYC ChIP on Nmp-1 promoter (positive control) in M1626 and HepG2 cells. [file Image3.tiff]
